# Ubiquitylation Directly Induces Fold Destabilization of Proteins

**DOI:** 10.1038/srep39453

**Published:** 2016-12-19

**Authors:** Daichi Morimoto, Erik Walinda, Harumi Fukada, Kenji Sugase, Masahiro Shirakawa

**Affiliations:** 1 Department of Molecular Engineering, Graduate School of Engineering, Kyoto University, Kyoto-Daigaku Katsura, Nishikyo-Ku, Kyoto 615-8510, Japan; 2 Department of Molecular and Cellular Physiology, Graduate School of Medicine, Kyoto University, Yoshida Konoe-cho, Sakyo-ku, Kyoto, 606-8501, Japan; 3 Center for Medical Education, Graduate School of Medicine, Kyoto University, Yoshida Konoe-cho, Sakyo-ku, Kyoto, 606-8501, Japan; 4 Graduate School of Life and Environmental Sciences, Osaka Prefecture University, Naka-ku, Sakai, Osaka 599-8531, Japan

## Abstract

Ubiquitin is a common post-translational modifier and its conjugation is a key signal for proteolysis by the proteasome. Because the molecular mass of ubiquitin is larger than that of other modifiers such as phosphate, acetyl, or methyl groups, ubiquitylation not only influences biochemical signaling, but also may exert physical effects on its substrate proteins by increasing molecular volume and altering shape anisotropy. Here we show that ubiquitylation destabilizes the fold of two proteins, FKBP12 and FABP4, and that elongation of the conjugated ubiquitin chains further enhances this destabilization effect. Moreover, NMR relaxation analysis shows that ubiquitylation induces characteristic structural fluctuations in the backbone of both proteins. These results suggest that the ubiquitylation-driven structural fluctuations lead to fold destabilization of its substrate proteins. Thus, physical destabilization by ubiquitylation may facilitate protein degradation by the proteasome.

Ubiquitylation is a common post-translational modification of physiological importance equivalent to phosphorylation, acetylation, and methylation. In this modification, ubiquitin is covalently conjugated to a lysine residue or the N-terminal residue of a substrate protein via its C-terminal tail^1^. The C-terminal tail of ubiquitin can also be covalently conjugated to a lysine residue or the N-terminal residue of another ubiquitin molecule, thereby forming polyubiquitin chains. (Poly-)ubiquitin molecules attached to a substrate protein are specifically recognized by down-stream ubiquitin-binding proteins^2^. One of the most well-known cellular processes associated with ubiquitylation is protein degradation^3^, in which polyubiquitin-tagged proteins are targeted to the 26S proteasome, where they are unfolded and degraded by the proteasome in an ATP-dependent manner^4^. Ubiquitylation also exerts non-proteolytic functions such as the regulation of protein activity and localization^1^. Thus, similar to other posttranslational modifications, ubiquitylation participates in many cellular processes by controlling protein function.

Ubiquitin (8.6 kDa) and ubiquitin-like modifiers (8–20 kDa)^5^ are relatively high-molecular weight entities, as compared with other post-translational modifiers such as acetyl (43 Da), methyl (15 Da), and phosphate (97 Da) groups. This suggests that conjugation of a ubiquitin molecule to a substrate protein might also affect some physical properties of the substrate such as molecular weight/volume and molecular shape anisotropy. Indeed, a recent molecular dynamics analysis showed that ubiquitylation might be capable of causing partial unfolding of substrate proteins^6^. Furthermore, we previously observed a decrease in the thermodynamic stability of ubiquitin itself due to polymerization^7^. We therefore hypothesized that the fold of ubiquitylated proteins might be destabilized via a molecular mechanism similar to that observed for polyubiquitin chains. Furthermore, the ubiquitylation-induced destabilization of substrate proteins might lead to the formation of aggregates or might shorten their intracellular lifetime.

## Results

**Figure 1:**
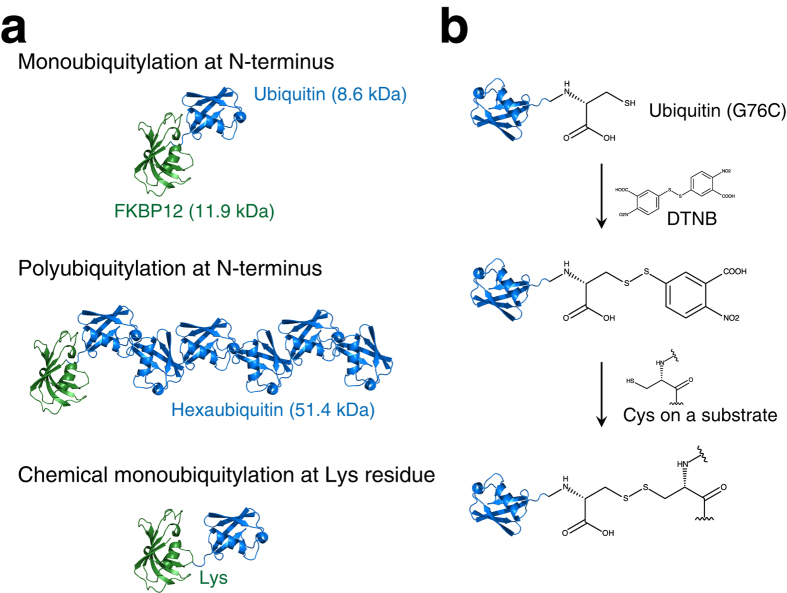
Schematic diagrams of ubiquitylation and synthesis of chemically ubiquitylated proteins. (**a**) The three types of ubiquitylation examined in the study. (**b**) Scheme for chemical conjugation of ubiquitin to a substrate protein via a disulfide bond.

We first prepared ubiquitylated proteins with two distinct kinds of linkage between ubiquitin and the target protein: N-terminal ubiquitylation^8^ and site-specific ubiquitylation by chemical conjugation at a site where intracellular ubiquitylation has been previously confirmed (Fig. 1a). We used two proteins that have been shown to be ubiquitylated *in vivo*: human FK506-binding protein (FKBP12) and human fatty acid binding protein 4 (FABP4). According to the PhosphoSite Plus database^9^, FKBP12 is ubiquitylated at Lys35, Lys36, Lys48, and Lys53; FABP4 is ubiquitylated at Lys22, Lys32, Lys38, Lys59, Lys80, Lys97, Lys101, Lys113, and Lys121. Each protein is composed of a single-domain structure; therefore, the physical effect of ubiquitylation on these proteins should be easy to compare. To obtain the N-terminally ubiquitylated form, we fused the gene encoding monoubiquitin (Ub) or tandemly arranged hexaubiquitin (Ub_6_) to the gene encoding each protein to express Ub-FKBP12, Ub_6_-FKBP12, Ub-FABP4, and Ub_6_-FABP4. To prepare chemically ubiquitylated proteins, we constructed a simple and efficient ubiquitylation protocol using disulfide conjugation. By activating cysteine thiol groups with 5,5′-dithiobis-(2-nitrobenzoic acid) (DTNB)^10^, a disulfide bridge was formed specifically between the C-terminus of ubiquitin and the native ubiquitylation site of the substrate protein (Fig. 1b). This disulfide-mediated ubiquitylation has been shown to mimic native ubiquitylation^10^,^11^,^12^ although it contains an extra carboxyl group (Fig. 1b), implying that the disulfide-mediated ubiquitylation may not cover all the biochemical features of native ubiquitylation. However, in cases where ubiquitin-conjugating enzymes and ubiquitin ligases for target proteins are not identified, the disulfide-mediated ubiquitylation is the most straightforward way to prepare sufficient amounts of ubiquitylated protein samples for thermodynamic analysis. In this study, we prepared chemically ubiquitylated FKBP12 at Lys36, Lys48, and Lys53; and chemically ubiquitylated FABP4 at Lys32, Lys80, and Lys121. Each ubiquitylation site is located in a characteristic secondary structure element: namely, a loop, α-helix, or β-sheet (Supporting Information (SI), Figure S1).

**Figure 2:**
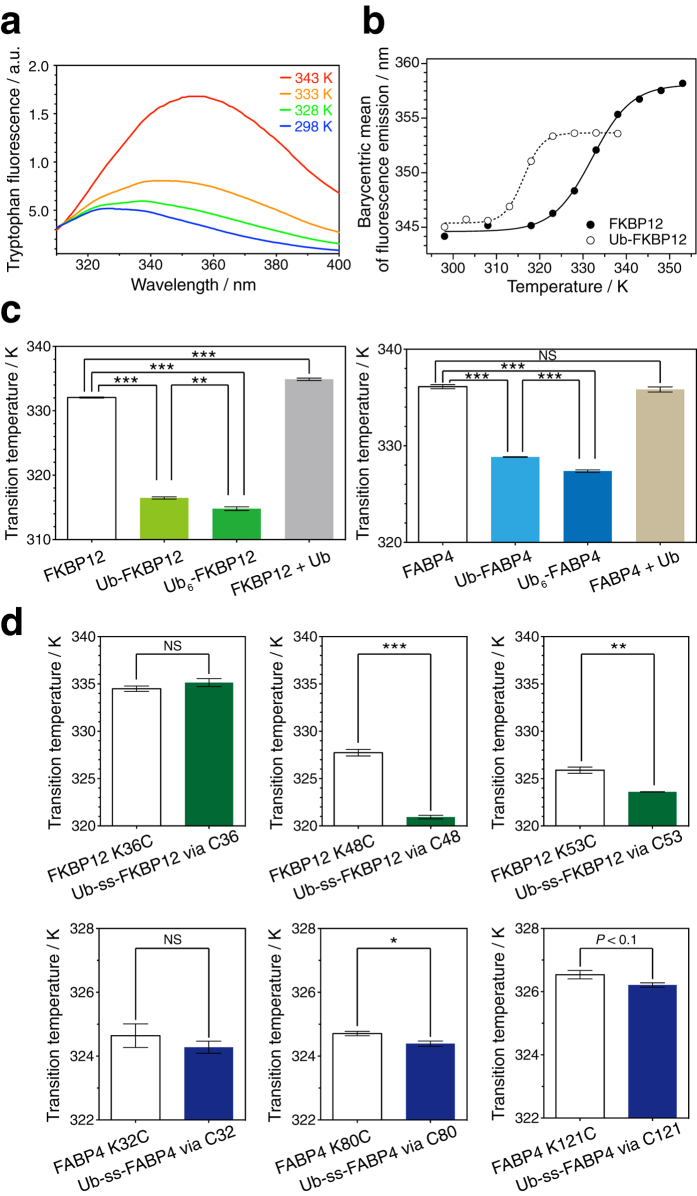
Fold destabilization of a substrate proteins by (poly-)ubiquitylation. (**a**) Temperature-dependent changes in the tryptophan fluorescence emission spectra of FKBP12. The emission maximum in the fluorescence spectra shows a red shift with increasing temperature. (**b**) Series of fluorescence emission wavelengths (barycentric mean) of FKBP12 (filled circles) and N-terminally mono-ubiquitylated FKBP12 (open circles). The data were fitted to the sigmoidal equation (solid and dashed line, respectively). (**c**) Comparative analysis of the thermal denaturation transition for N-terminal (poly-)ubiquitylated FKBP12 (left) and FABP4 (right). Simple addition of ubiquitin to a substrate protein had little effect on its thermostability. (**d**) Comparative analysis of thermal denaturation transitions for chemically ubiquitylated FKBP12 (upper) and FABP4 (lower). For disulfide conjugation, FKBP12 and FABP4 carry the additional mutations C22S and C2A/C118A, respectively. ss indicates the disulfide bridge. All values represent the average of three independent experiments. Error bars indicate the standard error of the mean. ***P < 0.001, **P < 0.01, and *P < 0.05 (Student’s *t* test). NS indicates no statistical significance: *P* > 0.1. Transition temperature values and statistics are shown in Table 1.

To investigate the physical effect of ubiquitylation, we compared the fold stability of the two ubiquitylated proteins with that of the non-ubiquitylated form. Native ubiquitin has no tryptophan residues, whereas both substrate proteins (FKBP12 and FABP4) have several. As a result, the tryptophan fluorescence spectrum of the ubiquitylated protein specifically reflects the chemical environment of tryptophan residues in the substrate proteins. Furthermore, when a tryptophan residue participates in hydrogen bonding and/or is exposed to water, its fluorescence emission shifts to longer wavelengths; therefore, the emission wavelength changes in response to conformational changes in the substrate proteins (*e*.*g*., heat-denaturation of FKBP12; Fig. 2a). Tryptophan fluorescence analysis indicated that N-terminal mono-ubiquitylation decreased the thermal transition temperature of both substrate proteins by more than 5 K (Fig. 2b and c). Such ubiquitylation-induced fold destabilization was also observed when inducing chemical denaturation using guanidine hydrochloride (SI, Figure S2). In stark contrast, a simple mixture of protein and ubiquitin (without covalent conjugation) did not destabilize the fold of those proteins (Fig. 2c and SI, Figure S3). In particular, the mixture of ubiquitin molecules stabilized the fold of FKBP12. Non-specific weak protein interactions between ubiquitin and FKBP12 might contribute to this thermodynamic stabilization although such an interaction was undetectable in NMR titration experiments (data not shown). Interestingly, N-terminal poly-ubiquitylation (attachment of linear hexaubiquitin) further enhanced the unfolding of the substrate proteins to a limited but further extent (*ca*. 1.5 K) (Fig. 2c and SI, Figure S3). Similar to N-terminal ubiquitylation, site-specific chemical ubiquitylation decreased the transition point of both substrate proteins (Fig. 2d and SI, Figure S4). As compared with N-terminal ubiquitylation, the effect of chemical ubiquitylation on substrate fold stability seemed to be smaller. Intriguingly, the degree of fold destabilization appeared to depend on secondary structure elements of the substrate protein at the site of ubiquitylation (SI, Figure S1). Ubiquitylation at a site in a β-sheet (Fig. 2d middle) caused larger fold destabilization than that in loops (Fig. 2d right). On the other hand, ubiquitylation at a site in an α-helix had no significant effect on fold stability of FABP4 (Fig. 2d lower left). Fold destabilization was not observed for FKBP12 ubiquitylated at Cys36 (Fig. 2d upper left), which is located in a short β-sheet. Taken together, these results indicate that covalent conjugation of (poly-)ubiquitin to the substrate protein decreases the fold stability of the substrate and the degree of destabilization depends on the chain length and the site of ubiquitylation.

**Figure 3:**
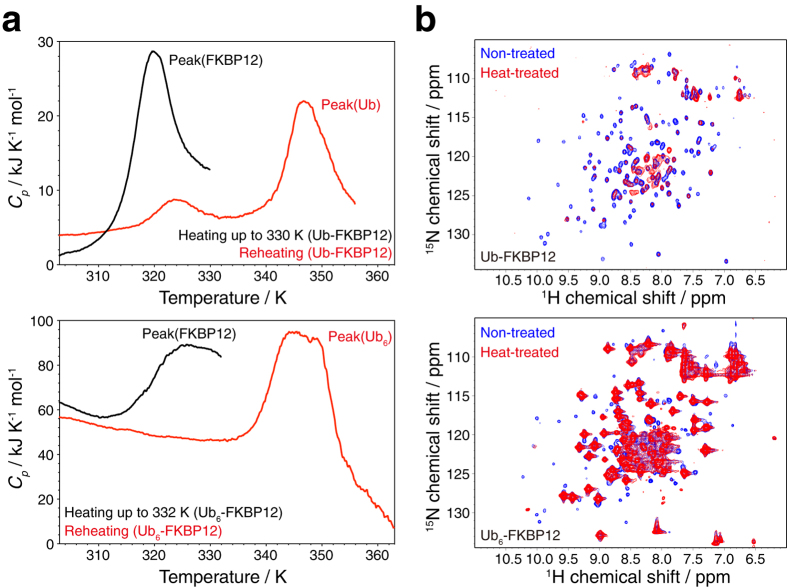
Irreversible thermal unfolding of FKBP12 in its N-terminally ubiquitylated form. (**a**) Differential scanning calorimetry (DSC) traces of Ub-FKBP12 (upper) and Ub_6_-FKBP12 (lower). Black traces show initial heating of Ub-FKBP12 to 330 K (upper) and Ub_6_-FKBP12 to 332 K (lower); red lines show respective reheating of the same samples. Peak assignments were based on previous DSC measurements of FKBP12 and (poly-)ubiquitin^7^. (**b**) ^1^H-^15^N HMQC spectra of non-heated (blue) and heated (335 K) (red) ubiquitylated FKBP12. Upper, 40 μM Ub-FKBP12 at 298 K; lower, 48 μM Ub_6_-FKBP12 at 310 K. Most of the peaks observed for folded FKBP12 were not detected in the spectrum of heat-treated ubiquitylated FKBP12; however, (poly-)ubiquitin remained folded after heat treatment.

We previously observed that the thermal unfolding of FKBP12 is reversible; in contrast, N-terminally ubiquitylated FKBP12 displays an irreversible transition^7^. Similarly, ubiquitin loses its thermal folding reversibility by polymerization or conjugation to other proteins^7^; thus, we considered whether FKBP12 in its ubiquitylated form is simply entrapped by insoluble ubiquitin aggregates during heat denaturation, or whether it loses its own folding reversibility alongside ubiquitin. To probe the folding reversibility of FKBP12 in ubiquitylated form, we monitored its differential scanning calorimetry (DSC) profile after selective thermal unfolding of FKBP12. Because the thermal transition point of FKBP12 is approximately 20 K lower than that of (poly-)ubiquitin, it is possible to denature only the FKBP12 moiety in Ub-FKBP12 or Ub_6_-FKBP12, while leaving ubiquitin folded. Although the transition point of free FKBP12 was found to be 335.5 K in previous DSC measurements^7^, we observed a DSC peak at a position 10 K lower than the transition of free FKBP12, which we assumed corresponds to the transition of FKBP12 in its (poly-)ubiquitylated form^7^. This observation is consistent with the thermodynamic destabilization of FKBP12 by N-terminal ubiquitylation observed in the tryptophan fluorescence experiments (Fig. 2c). After Ub-FKBP12 was heated to 330 K and gently cooled to room temperature, the DSC peak corresponding to FKBP12 was barely observed in the reheating thermograph. However, the peak of ubiquitin was readily detected (Fig. 3a, upper). Similar to Ub-FKBP12, the DSC peak corresponding to FKBP12 could not be detected in the reheating thermograph of Ub_6_-FKBP12, whereas that of linear hexaubiquitin was detected (Fig. 3a, lower). These results indicate that (poly-)ubiquitylation abolishes the thermal folding reversibility of the modified protein. Interestingly, heat-treated (poly-)ubiquitylated FKBP12 was soluble, and DSC analysis indicated that heat denaturation of FKBP12 did not affect the folding of (poly-)ubiquitin[Table t1].

To investigate what structural changes occur during the heat denaturation of FKBP12 in (poly-)ubiquitylated FKBP12, we measured its ^1^H-^15^N hetero-nuclear multiple quantum coherence (HMQC) spectrum. The spectrum of non-heated (poly-)ubiquitylated FKBP12 displayed peaks of both FKBP12 and ubiquitin in positions corresponding to approximately the sum of those of the unconjugated protein and modifier. In contrast, no peaks corresponding to natively folded FKBP12, but peaks of (poly-)ubiquitin were detected in the spectrum of (poly-)ubiquitylated FKBP12 that had been heat-treated up to 330 K or 332 K (Fig. 3b and SI, Figure S5). Instead, there were several new peaks, positioned mainly between 8.5 and 8.0 ppm in the ^1^H dimension—a region where peaks are often observed for unstructured proteins/peptides. This result implies that the FKBP12 protein in (poly-)ubiquitylated FKBP12 is irreversibly unfolded by the heat treatment up to 330 K or 332 K, but the ubiquitin moiety is not.

To gain insight into the mechanism of protein destabilization by ubiquitylation, we prepared ubiquitylated proteins in which only the substrate proteins were ^15^N-labeled in order to selectively observe NMR peaks of the substrate protein, but not those of the attached ubiquitin, in ^1^H-^15^N hetero-nuclear single quantum coherence (HSQC) spectra. The spectra of ubiquitylated FKBP12 and FABP4 showed little chemical shift perturbation as compared with the respective non-ubiquitylated proteins (SI, Figure S6). This observation indicates that ubiquitylation does not significantly change the overall average structure of a given substrate protein at moderate temperature.

**Figure 4:**
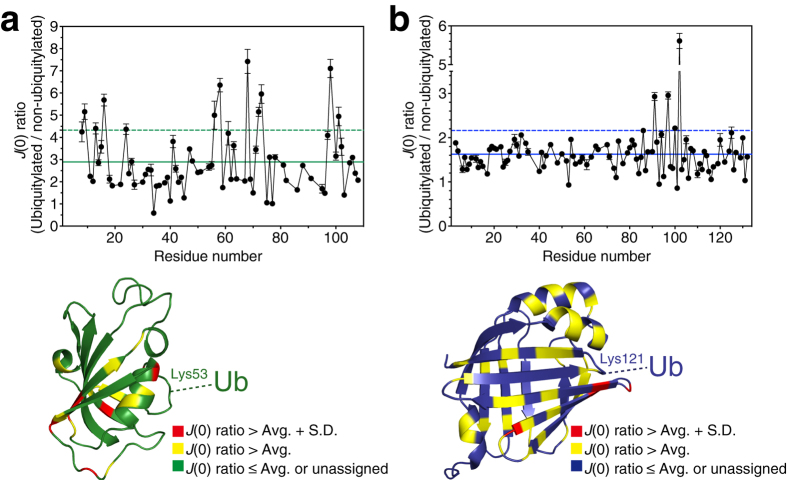
Increased in structural fluctuations in substrate proteins due to ubiquitylation. Upper, spectral density function *J*(0) ratio of the ubiquitylated to the non-ubiquitylated form of FKBP12 (**a**) and FABP4 (**b**). Solid and dashed lines indicate the average and standard deviation of the *J*(0) ratio, respectively. Lower, mapping of the *J*(0) ratio onto the individual structures (PDB database accession codes: 2PPN for FKBP12^29^ and 3RZY for FABP4)^30^. The respective ubiquitylation site is indicated.

Next, we analyzed the effect of ubiquitylation on protein backbone dynamics at three different frequencies by deriving spectral density functions, *J*(0), *J*(ω_N_), and *J*(0.87ω_H_), from the ^15^N *T*_1_, *T*_2_ relaxation times, and steady-state {^1^H}-^15^N heteronuclear NOE values (SI, Figure S7). The spectral density functions quantitatively report dynamics on a variety of timescales (from pico- to milliseconds)^13^. We observed more diversity in the spectral density functions of both ubiquitylated proteins as compared with their non-ubiquitylated counterparts (with the exception of *J*(ω_N_) of ubiquitylated FKBP12) (SI, Figure S8). The spectral density functions of both of the non-ubiquitylated proteins were relatively uniform across residues. The most marked difference in function was observed for *J*(0): the mean ± standard deviation values for FKBP12 in the non-ubiquitylated and ubiquitylated form were 4.2 ± 1.3 ns and 11.4 ± 5.2 ns, respectively; those for FABP4 were 5.2 ± 1.8 ns and 8.0 ± 2.2 ns, respectively. The larger values and deviations of *J*(0) observed for the ubiquitylated proteins versus non-ubiquitylated proteins may be partly due to the larger molecular mass and more anisotropic shape of ubiquitylated proteins. However, the magnitude of these differences seems to be beyond what might be assumed by differences in molecular weight and shape anisotropy, in particular for FKBP12 (Fig. 4). *J*(0) is sensitive to slow protein motions (on the micro- to millisecond timescale), including partial protein folding–unfolding. Thus, the larger values and deviations of *J*(0) observed for the ubiquitylated proteins might be caused by fluctuations in the protein backbone. ^15^N relaxation dispersion experiments for the ubiquitylated proteins suggested that ubiquitylation affected their intrinsic millisecond timescale protein motions to a certain degree (SI, Figures S9 and S10). In addition, rotational diffusion analysis of the observed relaxation parameters (^15^N *T*_1_, *T*_2_ relaxation times, and ^1^H-^15^N hetero-nuclear NOE values) using the program *ROTDIF*^14^ showed ubiquitylation-induced differences in rotational diffusion anisotropy for the modified protein (SI, Figure S11 and Table S1). The conjugation of ubiquitin resulted in an exchange of isotropic rotational diffusion to an anisotropic one for FKBP12 and a directional change of the rotational diffusion tensor for FABP4 (SI, Figure S11 and Table S1). Taken together, these observations suggest that the intrinsic protein motion of the substrate protein is disturbed by ubiquitylation, and that the resulting increase in global fluctuations may lead to decreased stability of the structural fold of that protein.

## Discussion

In this study, we present experimental evidence for the ubiquitylation-induced destabilization of the structural fold of proteins. For both of the substrate proteins examined, covalent conjugation of (poly-)ubiquitin led to a decrease in the temperature of the thermal unfolding of the protein. We also observed that (poly-)ubiquitylation abolishes the thermal folding reversibility of FKBP12 (Fig. 3). When protein unfolding is thermally irreversible, it is difficult to obtain the Gibbs free energy difference between its folded and unfolded states from the thermal denaturation experiments. On the other hand, in such a case, the transition midpoints depend on the kinetics of the transitions^15^,^16^. Because all our fluorescence experiments were performed in the same manner, it is possible to compare the stabilities of the protein fold (fold stabilities) using the transition midpoints. In addition, the ubiquitylation-induced destabilization of FKBP12 was observed in the chemical denaturation experiments using guanidine chloride (SI, Figure S2), where no aggregation occurred. Thus, it is most likely that the ubiquitylation-induced fold destabilization could be detected in our experiments.

The degree of this ubiquitylation-induced fold destabilization depends on the modification site in the substrate protein (Fig. 2c and d). Both FKBP12 and FABP4 possess β-sheets at their N-terminal regions and their ubiquitylation at another β-sheet affected fold stability more severely than ubiquitylation in a loop or α-helix (Fig. 2d). Thus, secondary structure elements at ubiquitylation sites may be correlated with the degree of resultant destabilization. Because ubiquitin attachment is also used as a non-proteolytic intracellular signal, it is reasonable that the degree of fold destabilization varies in accordance with the ubiquitylation site. On the other hand, the ubiquitylation-induced structural fluctuations were not only located at the ubiquitylation site but also distributed rather globally. It will therefore be necessary to further investigate the mechanism underlying the structural fluctuations and fold destabilization caused by ubiquitylation.

Intracellular proteins do not consist exclusively of single-domain structures, but also contain many multi-domain assemblies. For a multi-domain protein, there may be diverse interactions between domains and thermodynamic properties^17^,^18^, and it may be complicated to estimate the effect of ubiquitylation on its fold stability. On the other hand, the average size of a single protein domain is approximately 100 residues^19^, which is approximately the size of the model proteins investigated in this study: FKBP12 and FABP4. This suggests that, in general, ubiquitylation is at least capable of causing fold destabilization of conjugated protein domain units. Indeed, in support of our hypothesis, we also observed a decrease in the thermodynamic stability of a multi-domain protein by mono-ubiquitylation: Ca^2+^ -free calmodulin (SI, Figure S12). It will be intriguing to examine how domain-domain interactions affect ubiquitylation-driven fold destabilization for multi-domain proteins that form more complicated quaternary structures.

On the one hand, a previous molecular dynamics analysis showed that ubiquitylation might stabilize the unfolded state of the substrate protein and that the resultant loss in folding entropy might cause its thermal instability^6^. On the other hand, the present study reveals that ubiquitylation induces changes in the dynamics of the folded state of the substrate protein, including structural fluctuations of the protein backbone. Such backbone fluctuations would directly affect the fold stability and, in fact, conjugation of ubiquitin was found to result in thermal destabilization of the substrate proteins even though it induces few static structural differences (Fig. 2 and SI, Figure S6). This discussion is consistent with a recent computational study, which showed that a ubiquitylation-induced increase in entropy in the folded state of the substrate protein causes thermodynamic destabilization^20^. Furthermore, the induction of more anisotropic molecular motion by ubiquitylation may be a possible factor that causes these changes in the structural dynamics of the substrate proteins. It will be important to identify the physical factors that might generate such structural changes and examine their related mechanisms.

During the course of proteasomal degradation, (poly-)ubiquitin-tagged proteins recruited to the 26S proteasome are unfolded by the AAA + ring of the proteasome in a process driven by ATP hydrolysis^21^. The protein is subsequently translocated to the proteasomal core, where it is degraded by the core protease machinery of the proteasome. Some intracellular proteins have degron sequences and/or intrinsically disordered tails/regions^22^, which trigger unfolding of the global structure; however, a substantial fraction of proteins (about 25%) have few such degrons. When a protein lacking a sufficient amount of such disordered tails/loops is targeted for degradation by the proteasome, some additional steps or factors to unfold that protein are needed. Therefore, we hypothesize that the direct partial unfolding by ubiquitylation might be one of the mechanisms that assist in the step of substrate unfolding. This direct partial unfolding by ubiquitylation would work favorably because it would decrease the amount of ATP required for the proteasome to achieve complete denaturation of a protein. Future studies will need to focus on elucidating the underlying mechanism and on examining the effect of multi-ubiquitylation of proteins.

Furthermore, the present results also suggest that the physical effects of ubiquitylation are related to intracellular aggregate formation. If (poly-)ubiquitin tagged proteins are not degraded by the proteasome appropriately or their ubiquitin molecules are not cleaved off efficiently, they will accumulate in cells. Given that polyubiquitin fibril formation is caused by the elongation of ubiquitin chains^7^, a poly-ubiquitylated protein may form irreversibly insoluble aggregates either via the chain-length-dependent destabilization of the polyubiquitin chain^7^ or via the ubiquitylation-induced destabilization of the substrate protein. This indicates that ubiquitylation is closely associated with intracellular protein aggregation. The relationship between ubiquitylation and human proteinopathies should be further elucidated in future studies.

## Methods

### Protein preparation

Human ubiquitin, human 12-kDa FK506-binding protein (FKBP12) and human fatty acid binding protein 4 (FABP4), and their cysteine mutants were expressed in *Escherichia coli* strain BL21 (*DE3*) grown in LB or M9 minimal media containing 99% ^15^N-labeled ammonium chloride (Cambridge Isotope Laboratories). Human ubiquitin was purified by cation exchange and size-exclusion chromatography. Human FKBP12 was expressed as a fusion protein with an N-terminal glutathione *S*-transferase and small ubiquitin-like modifier protein (GST-SUMO-1) tag. After cleavage of the tag by GST-SENP2 protease, FKBP12 was further purified by size-exclusion chromatography. Human FABP4 was expressed as a fusion protein with an N-terminal hexa-histidine (His_6_) SUMO-1 protein tag. After cleavage of the His_6_-SUMO tag by GST-SENP2 protease, FABP4 was further purified by size-exclusion chromatography. N-terminally ubiquitylated FKBP12 and FABP4 were expressed as fusion proteins with an HRV3C-cleavable C-terminal His_6_-tag and purified by Ni-NTA affinity chromatography. After cleavage of the C-terminal His_6_-tag by HRV3C protease, the proteins were further purified by anion exchange and size-exclusion chromatography.

### Chemical conjugation of ubiquitin to substrate proteins

A ubiquitin G76C mutant protein was reduced with 5 mM 2-mercaptoethanol, and then buffer-exchanged into 50 mM sodium phosphate pH 7.5 using a PD-10 desalting column (GE Healthcare). The reduced ubiquitin mutant was mixed with a 20-fold molar excess of 5,5′-dithiobis-(2-nitrobenzoic acid) (DTNB, Tokyo Chemical Industry) and then incubated for 1–3 hours at room temperature with vigorous shaking. The reaction solution was buffer-exchanged into ligation buffer (20 mM Tris-HCl, 50 mM NaCl and 1 mM EDTA, pH 7.0) using a PD-10 desalting column. The cysteine mutants of the respective substrate proteins were also reduced, buffer-exchanged into ligation buffer, and mixed with a 3-fold molar excess of activated ubiquitin G76C-DTNB for 1 hour.

### Isolation of chemically ubiquitylated proteins

Chemically ubiquitylated proteins were isolated by hydrophobic interaction chromatography (HIC) using a HiTrap Phenyl HP column (GE Healthcare). The proteins were dissolved in high salt buffer (2 M potassium phosphate, pH 7.0), applied to the column, washed with more than two column volumes, and eluted by a salt gradient (2 to 0 M potassium phosphate, pH 7.0 in five column volumes). The purity of the eluted proteins was verified by SDS-PAGE and MALDI-TOF mass spectrometry (Bruker) analysis.

### Fluorescence spectroscopy

Fluorescence was quantified on a FluoroMax4 (HORIBA) spectrometer. Tryptophan fluorescence was selectively measured by excitation at 300 nm, and emission spectra were collected over wavelengths of 310 to 400 nm with the slit width set at 5 nm. All samples were diluted to a final substrate protein concentration of 20 μM in PBS buffer. In the case of (Met1-ubiquitylated-)FKBP12 and FABP4, 0.5 mM TCEP was included in the buffer. The spectral contribution of the buffer was subtracted from the acquired spectra. The peak shift was evaluated by calculating the barycentric mean of the fluorescence emission spectrum. The barycentric mean was obtained from the equation 
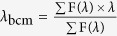
, in which F (λ) is the tryptophan fluorescence intensity at λ nm. Using Igor Pro 6 (WaveMetrics), transition points were obtained by fitting a series of tryptophan emission wavelengths to the sigmoid equation 
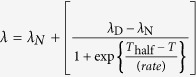
, in which λ is the tryptophan fluorescence emission wavelength observed at temperature T in units of Kelvin, and λ_N_, and λ_D_ are those of the native and the completely denatured proteins, respectively; T_half_ is the midpoint temperature of the sigmoidal curve; the *rate* is a parameter that determines the slope of the curve.

### Differential scanning calorimetry

Thermal denaturation curves were acquired on a Nano DSC instrument (TA Instruments Inc.). The scan rate was 1 K min^−1^, and the protein concentration was 1 mg ml^−1^. The buffer was PBS (137 mM NaCl, 8.1 mM Na_2_HPO_4_, 2.68 mM KCl and 1.47 mM KH_2_PO_4,_ pH 7.4) containing 0.1 mM TCEP. Reheating experiments were performed in the same manner after heating the protein to the target temperature, followed by gradual cooling to room temperature. Analysis was performed by using CpCalc (TA Instruments Inc.) and data were reported as heat capacity (kJ K^−1^ mol^−1^). The transition temperature was defined as the temperature corresponding to the transition peak maximum.

### NMR spectroscopy

All NMR spectra were acquired at 298 K or 310 K on a Bruker Avance 600 MHz NMR spectrometer equipped with a 5 mm ^15^N/^13^C/^1^H z-gradient triple resonance cryoprobe. Resonance assignments for ^1^H-^15^N peaks were based on previous studies^23^,^24^. To probe the folding of native and heat-treated (poly-)ubiquitylated FKBP12, ^1^H-^15^N SOFAST-HMQC^25^ spectra were acquired. Because (non-)heated ubiquitylated FKBP12 appeared to be unstable, the NMR spectra were obtained in a short amount of time by SOFAST-HMQC. The measurement conditions for SOFAST-HMQC spectra were PBS buffer containing 5 mM EDTA and 1 mM DTT for Ub-FKBP12, PBS buffer for Ub_6_-FKBP12, and PBS buffer containing 0.5 mM TCEP for FKBP12. To examine the chemical shift differences caused by ubiquitylation, ^1^H-^15^N HSQC spectra were acquired in phosphate buffer (20 mM potassium phosphate, 5 mM KCl, 1 mM EDTA, 50 mM NaCl, 5 mM DTT, pH 6.8) for the two proteins examined. The ^15^N relaxation experiments were also performed in this phosphate buffer. For the ^15^N *T*_1_ relaxation experiment, a series of spectra with relaxation delays of 10, 20, 40, 180, 300, 500, and 1000 milliseconds were measured. For the ^15^N *T*_2_ relaxation experiment, a series of spectra with relaxation delays of 10, 30, 50, 70, 90, 110, and 150 milliseconds were acquired. For the ^1^H-^15^N NOE, *T*_1_, and *T*_2_ relaxation measurements, the recycle delay was set to 3–5 seconds to ensure that there was adequate longitudinal relaxation between acquisitions. Data processing was performed in NMRPipe^26^ and CCPN^27^.

### NMR relaxation analysis

In the ^15^N *T*_1_ and *T*_2_ relaxation experiments, the signal intensities *I*(*t*) of each peak with different relaxation delays *t* were fitted to the equation 
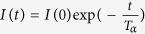
 to obtain the relaxation time *T*_α_, where α = 1 or 2. Fitting was performed using the program GLOVE^28^. ^1^H-^15^N heteronuclear NOE (hnNOE) values were calculated by the equation: (hnNOE value) = *I*_sat_ (*I*_eq_)^−1^, where *I*_sat_ and *I*_eq_ are the peak intensities with and without proton saturation, respectively. The values represent the average of two independent experiments. The respective spectral density functions, *J*(ω), were obtained from the ^15^N *T*_1_, *T*_2_ relaxation times, and hnNOE values^13^. Errors in the *T*_1_ and *T*_2_ relaxation times were calculated by the Monte Carlo method^28^, and errors in the hnNOE values were estimated by the standard deviation of two experiments. Errors bars for the spectral density functions were obtained by error propagation.

## Additional Information

**How to cite this article**: Morimoto, D. *et al*. Ubiquitylation Directly Induces Fold Destabilization of Proteins. *Sci. Rep.* **6**, 39453; doi: 10.1038/srep39453 (2016).

**Publisher's note:** Springer Nature remains neutral with regard to jurisdictional claims in published maps and institutional affiliations.

## Supplementary Material

Supplementary Information

## Figures and Tables

**Table 1 t1:** Transition temperatures and statistics for thermal denaturation.

Protein	Transition temperature/K	*P* value for Student’s *t* test
FKBP12	332.1 ± 0.1	—
Ub-FKBP12	316.5 ± 0.2	1.5 × 10^−7^
Ub_6_-FKBP12	314.8 ± 0.3	5.5 × 10^−7^; 7.8 × 10^−3^*
FKBP12 + Ub	334.9 ± 0.2	1.5 × 10^−4^
FKBP12^K36C^	334.5 ± 0.3	—
Ub-ss-FKBP12^C36^	335.1 ± 0.4	2.7 × 10^−1^
FKBP12^K48C^	327.7 ± 0.3	—
Ub-ss-FKBP12^C48^	320.9 ± 0.2	6.4 × 10^−5^
FKBP12^K53C^	325.9 ± 0.3	—
Ub-ss-FKBP12^C53^	323.6 ± 0.0	2.1 × 10^−3^
FABP4	336.1 ± 0.2	—
Ub-FABP4	328.8 ± 0.0	4.8 × 10^−6^
Ub_6_-FABP4	327.4 ± 0.1	3.9 × 10^−6^; 3.6 × 10^−4^*
FABP4 + Ub	335.8 ± 0.3	4.4 × 10^−1^
FABP4^K32C^	324.6 ± 0.4	—
Ub-ss-FABP4^C32^	324.3 ± 0.2	4.3 × 10^−1^
FABP4^K80C^	324.7 ± 0.1	—
Ub-ss-FABP4^C80^	324.4 ± 0.1	4.4 × 10^−2^
FABP4^K121C^	326.5 ± 0.1	—
Ub-ss-FABP4^C121^	326.2 ± 0.1	9.7 × 10^−2^

The superscripts show the point mutations or the site of disulfide bridge formation. The asterisks indicate the *P* values in Student’s *t* test between the transitions of mono- and hexaubiquitylated proteins.
