# Supplementary material for: Ubiquitylation Directly Induces Fold Destabilization of Proteins
Source: Sci Rep. 2016 Dec 19;6:39453. doi: 10.1038/srep39453 (PMC5172356; doi:10.1038/srep39453)
Supplement: Supplementary Information [file srep39453-s1.pdf]

Ubiquitylation Directly Induces Fold Destabilization of Proteins**

Daichi Morimoto, Erik Walinda, Harumi Fukada, Kenji Sugase, and Masahiro Shirakawa*

Supplementary Information

Supplementary Figures

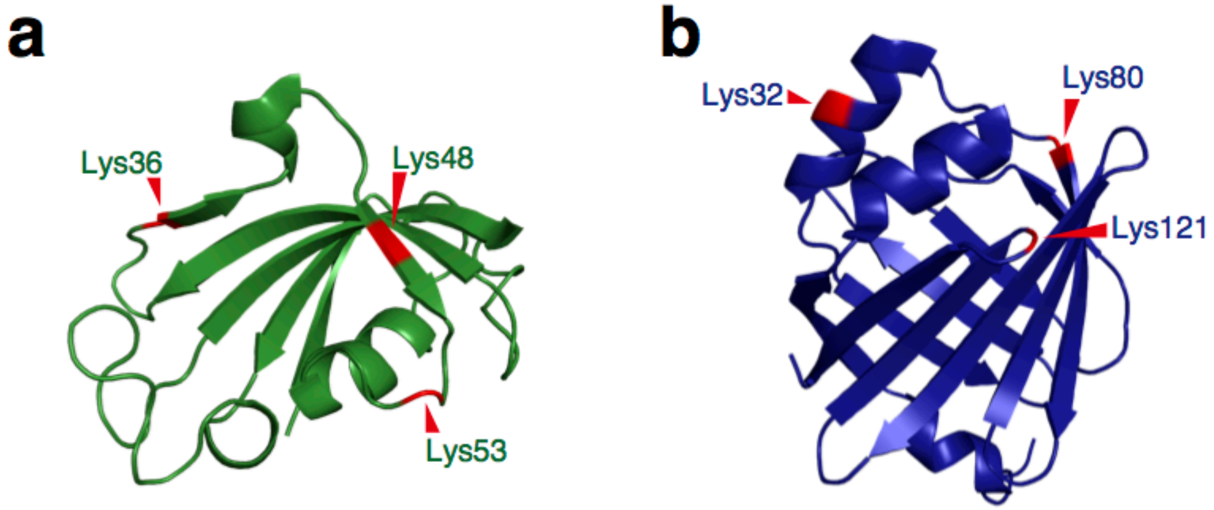

Figure S1. Ubiquitylation sites of FKBP12 and FABP4 examined. Each lysine residue colored in red is reported to be a ubiquitylation site according to the PhosphoSite Plus database. Each lysine residue was mutated to a cysteine residue to investigate the effect of ubiquitylation on the fold stability of the respective protein. In the structure of FKBP12 (**a**), Lys36, Lys48, and Lys53 are located in a short β-sheet, β-sheet, and loop, respectively. In the structure of FABP4 (**b**), Lys32, Lys80, and Lys121 are located in an α-helix, β-sheet, and loop, respectively. PDB database accession codes: 2PPN for FKBP12 and 3RZY for FABP4.

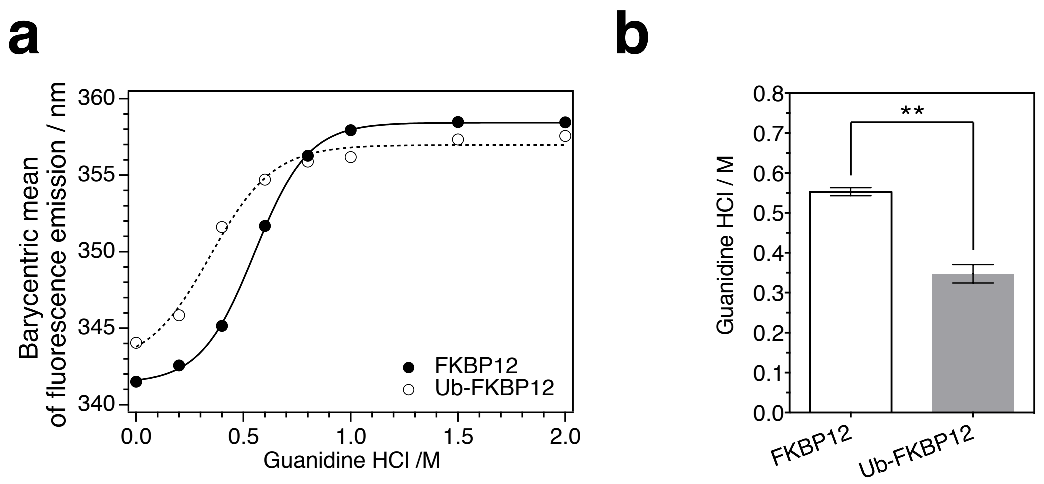

Figure S2. Examination of ubiquitylation-induced fold destabilization by chemical denaturation. a, Series of fluorescence emission wavelengths (barycentric mean) of FKBP12 (filled circles) and N-terminally mono-ubiquitylated FKBP12 (open circles). The data were fitted to a sigmoidal equation (See Methods): solid and dashed line, respectively. All samples were diluted to a final substrate protein concentration of 2 μM in PBS buffer. Guanidine hydrochloride (HCl) was used for the chemical protein denaturation. Data are representative of three independent experiments. **b**, Comparative analysis of the chemical denaturation transition for FKBP12 and N-terminally mono-ubiquitylated FKBP12. The transition for FKBP12 was 0.55 ± 0.01 M and that for mono-ubiquitylated FKBP12 was 0.35 ± 0.02 M. All values represent the average of three independent experiments. Error bars indicate the standard error of the mean. $^{**}P < 0.01$ (Student's *t* test).

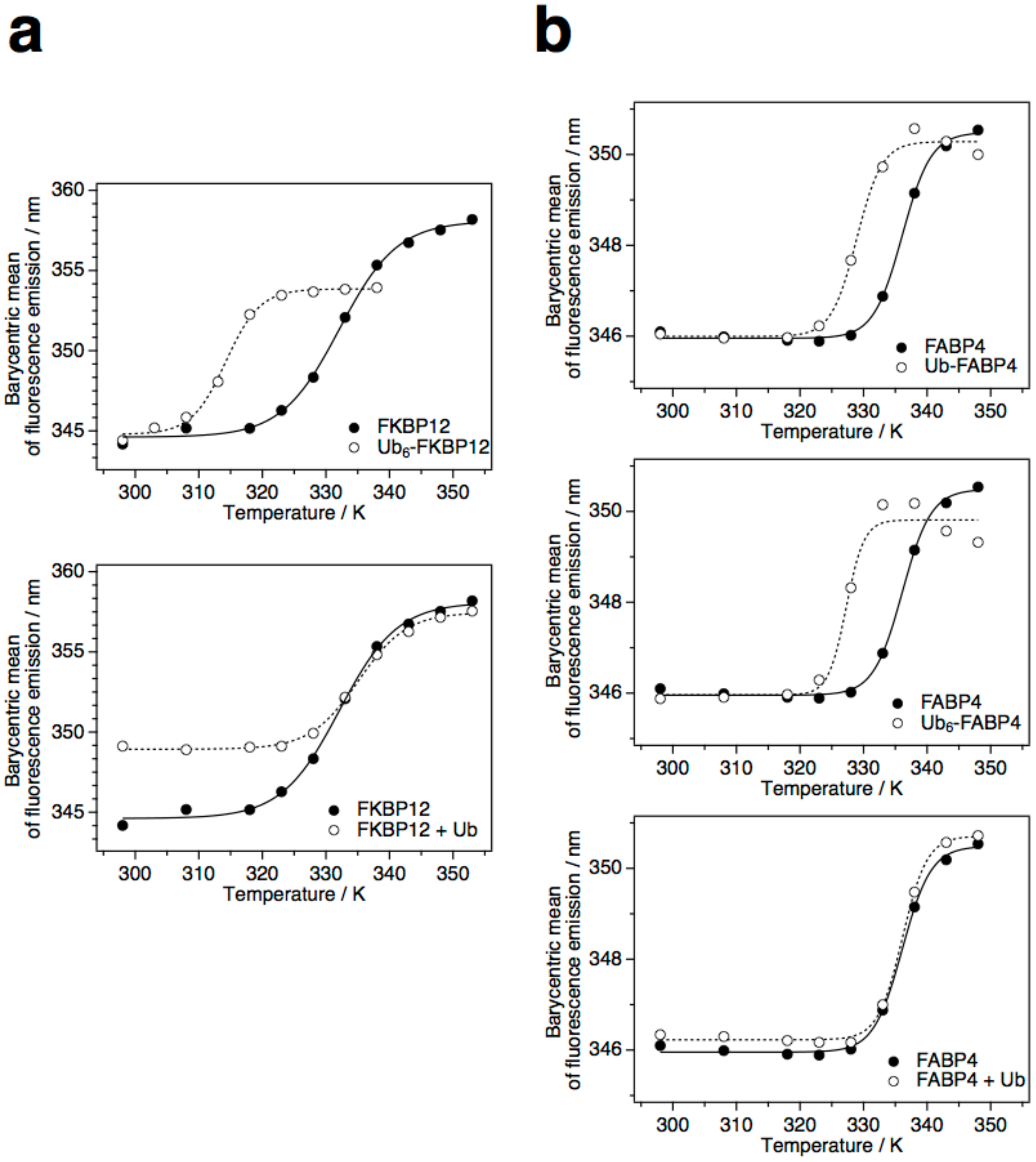

Figure S3. Temperature-induced red shift in the tryptophan fluorescence spectra of N-terminally (poly-)ubiquitylated proteins. The averaged fluorescence wavelength at each temperature was estimated by calculating the barycentric mean of the tryptophan fluorescence emission spectrum. The temperature-dependent fluorescence emission wavelengths of substrate proteins (filled circles), N-terminally (poly-)ubiquitylated substrate proteins (open circles) and substrate proteins mixed with ubiquitin (open circles) were fitted to the sigmoidal equation in the experimental section (FKBP12, **a**; FABP4, **b**). The profile for Ub-FKBP12 is shown in Figure 2b. Data are representative of three independent experiments.

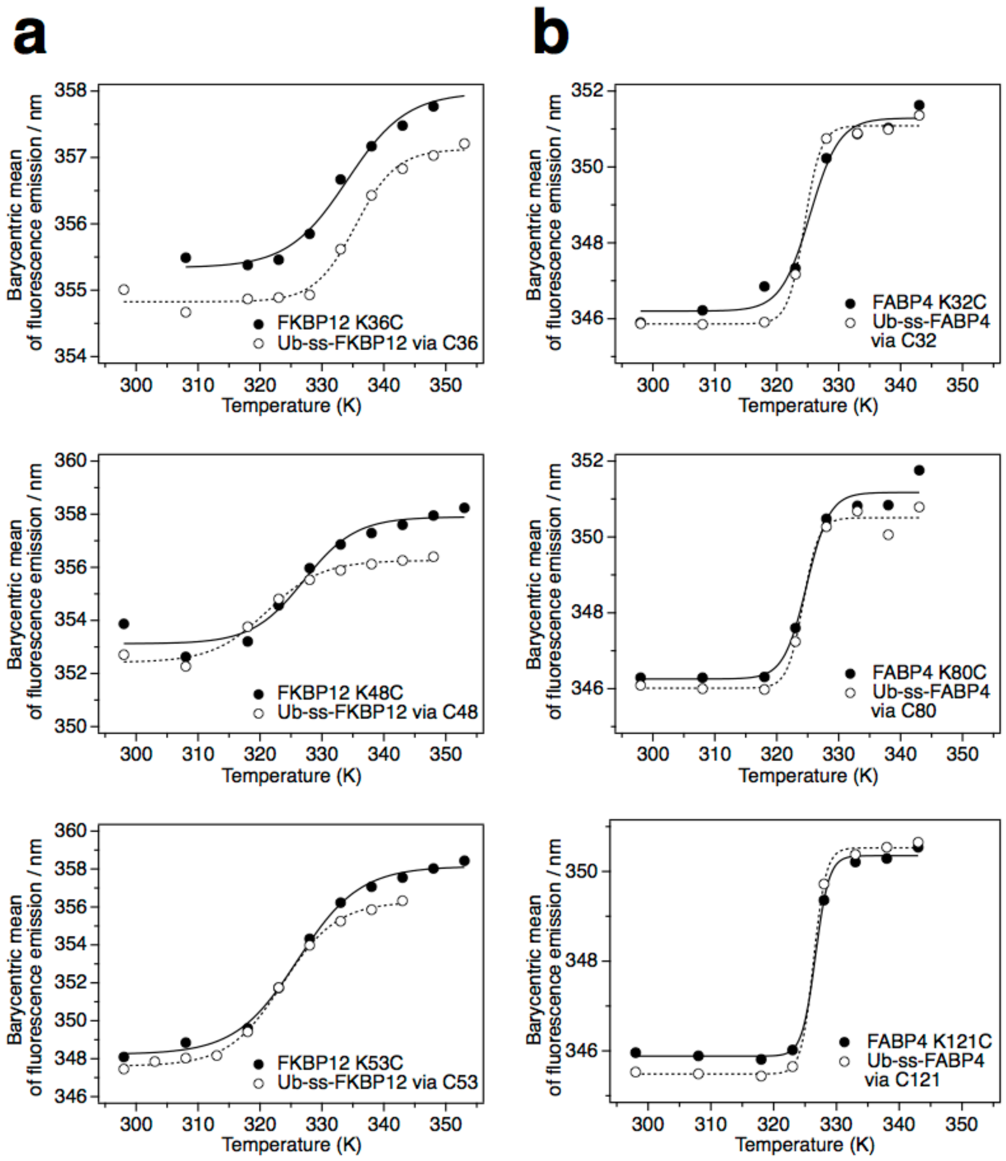

Figure S4. Temperature-induced red shift in the tryptophan fluorescence spectra of chemically ubiquitylated proteins. The averaged fluorescence wavelength at each temperature was estimated by calculating the barycentric mean of the tryptophan fluorescence emission spectrum. The temperature-dependent fluorescence emission wavelengths of substrate proteins (filled circles) and chemically ubiquitylated substrate proteins (open circles) were fitted to the sigmoidal equation in the experimental section (FKBP12, **a**; FABP4, **b**). The data point at 298K for FKBP12 K36C contained unexpected experimental noise and was removed from the analysis (**a**, top). Amino acid abbreviations are described in Figure 2d. Data are representative of three independent experiments.

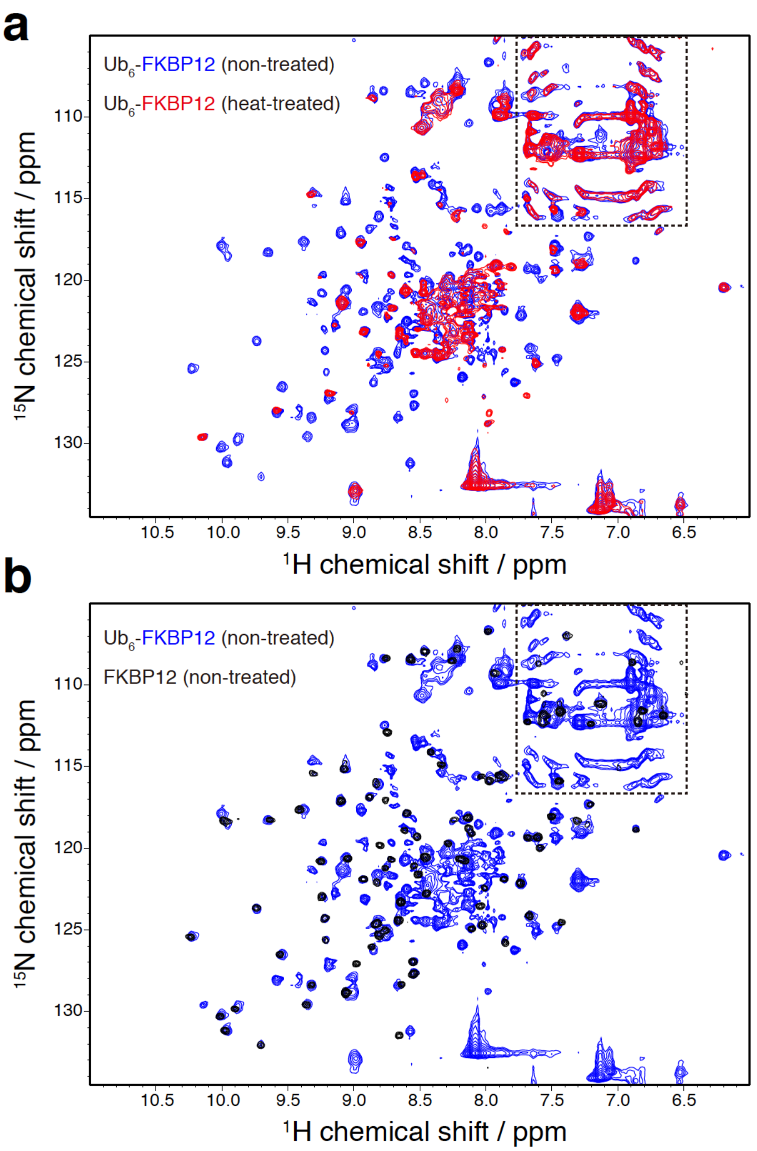

Figure S5. Irreversible thermal unfolding of FKBP12 in Ub_6-FKBP12. a, ^1H-^{15}N HMQC spectra of non-heated (blue) and heated (up to 332K, red) Ub_6-FKBP12. These spectra were obtained by subtracting the HMQC spectrum of Ub_6 from each spectrum of Ub_6-FKBP12. **b**, Comparative analysis of the spectrum of non-heated Ub_6-FKBP12 (blue) with that of non-heated FKBP12 (black). The region enclosed with dashed lines contains artificial signals arising from subtraction of the spectra.

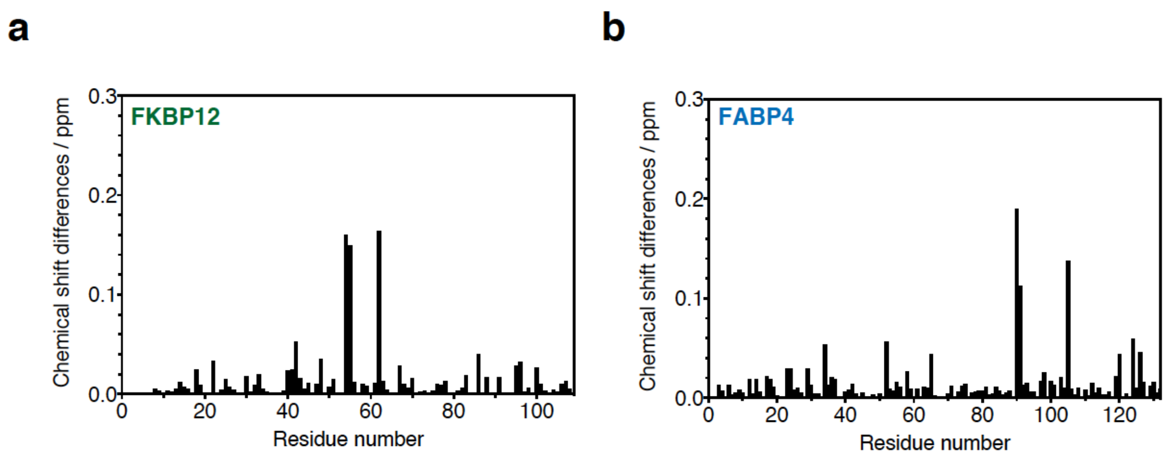

Figure S6. Ubiquitylation has little effect on the ground state structure of substrate proteins. Chemical shift perturbation due to chemical ubiquitylation of FKBP12 (**a**) and FABP4 (**b**), as obtained from the equation $\delta_{wt} = [\delta_{HN}^2 + (\delta_N / 5)^2]^{0.5}$ where δ_{HN} and δ_N are the ^{1}H and ^{15}N chemical shift changes between non-ubiquitylated and ubiquitylated proteins. Chemical shifts were obtained from the HSQC spectrum recorded for each 500 μM protein at 298 K.

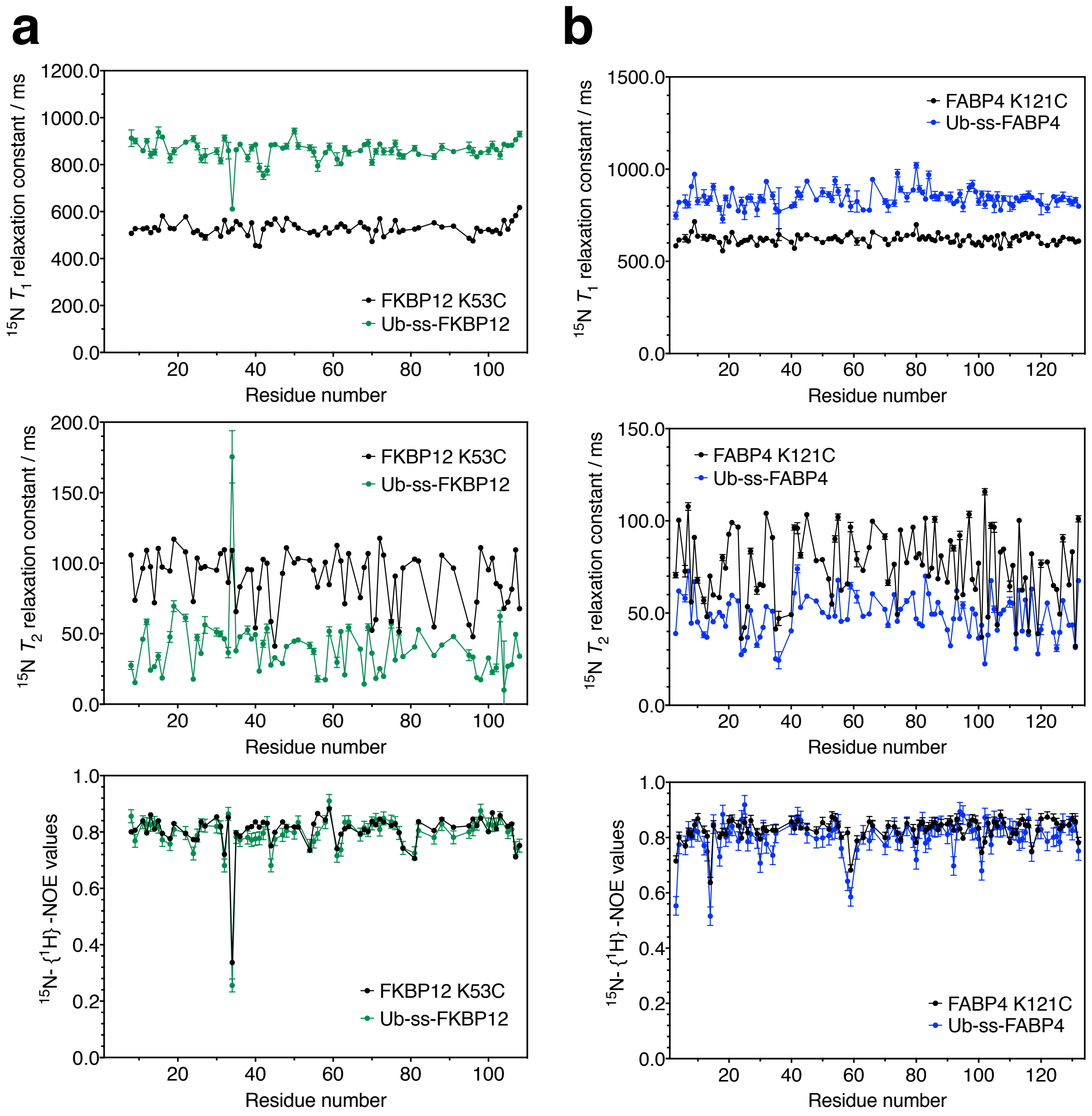

Figure S7. Relaxation times and hetero-nuclear NOE values of (non-)ubiquitylated proteins. Comparative analysis of ^{15}N T_1, T_2 relaxation times, and ^{1}H-^{15}N hetero-nuclear NOE values of non-ubiquitylated and ubiquitylated FKBP12 (**a**) or FABP4 (**b**). Parameters were obtained from analysis of the NMR spectrum of 500 µM (non-)ubiquitylated FKBP12 or 350 µM (non-)ubiquitylated FABP4 at 298 K. Amino acid substitutions are described in Figure 2d.

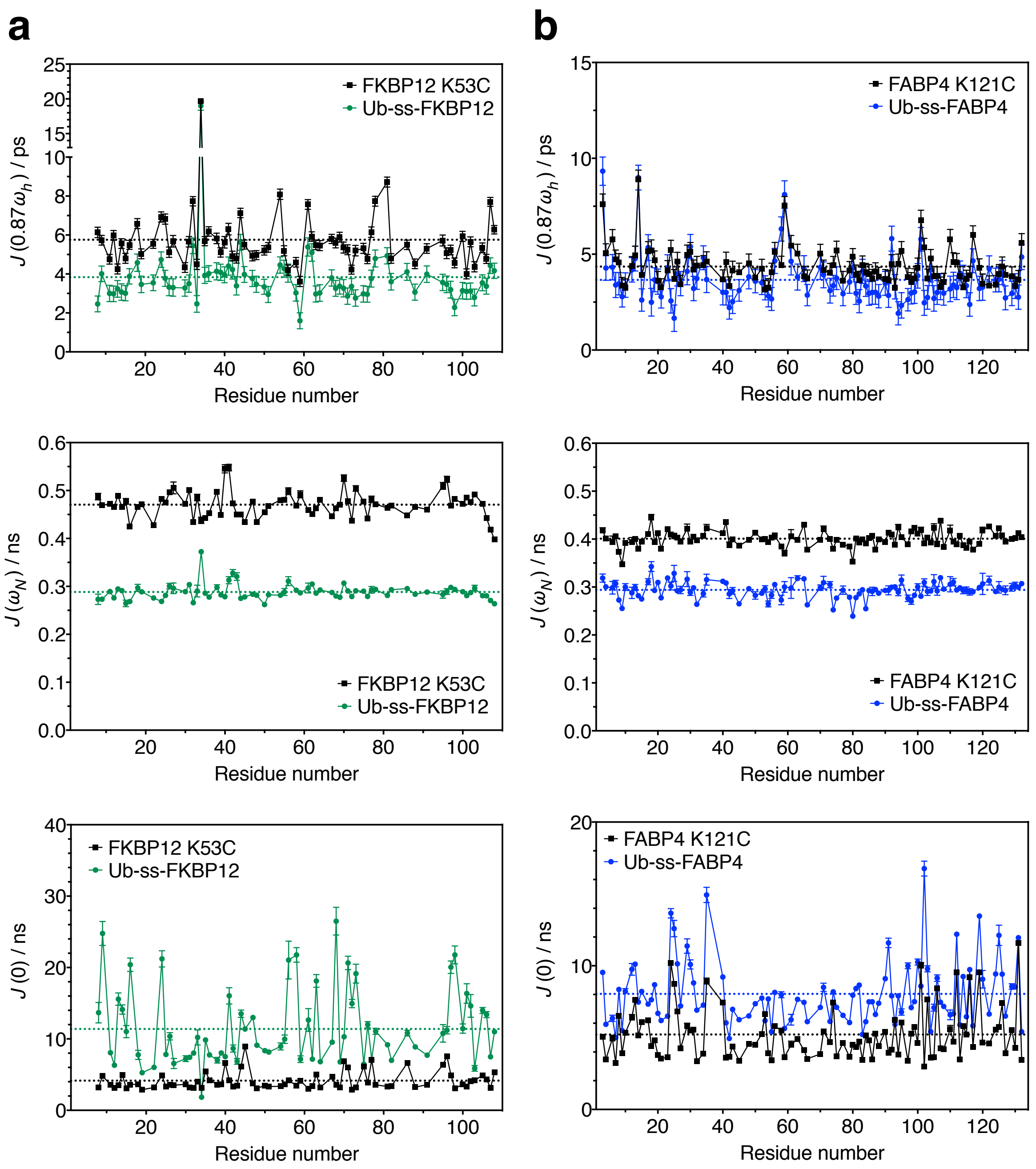

Figure S8. Spectral density functions at three different frequencies of (non-)ubiquitylated proteins. a, Comparative analysis of the $J(\omega)$ values for FKBP12 (black) and chemically ubiquitylated FKBP12 (green). **b**, Comparative analysis of the $J(\omega)$ values for FABP4 (black) and chemically ubiquitylated FABP4 (blue). Dashed lines indicate the average of the respective $J(\omega)$ values. Amino acid substitutions are described in Figure 2d.

a

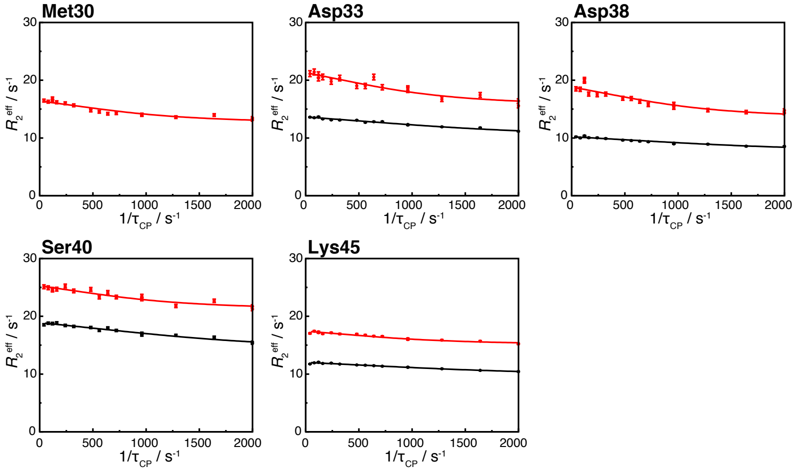

b

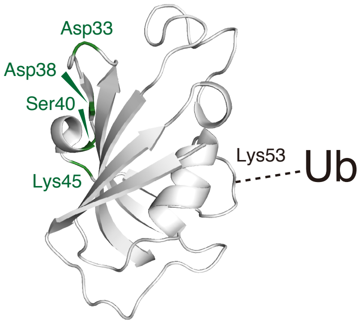

Figure S9. Relaxation dispersion analysis for ubiquitylated FKBP12. a, ^{15}N relaxation dispersion profiles for FKBP12 K53C (black) and chemically ubiquitylated FKBP12 via C53 (red). ^{15}N R_2 relaxation rates were measured by the Carr-Purcell-Meiboom-Gill (CPMG) pulse sequence[5]. The effective R_2 relaxation rate at a particular delay τ_{CP} was obtained from the equation: $R_2^{eff}\left(\tau_{CP}\right) = -\frac{1}{T_{CPMG}}\ln\left[\frac{I\left(\tau_{CP}\right)}{I_0}\right]$, in which τ_{CP} is a delay between two 180° pulses in the CPMG pulse sequence, T_{CPMG} is a fixed relaxation time, $I(\tau_{CP})$ is peak intensity at a delay τ_{CP}, and I_0 is the peak intensity in the reference spectrum. Respective data were globally fitted to the Luz and Meiboom equation[6]. The obtained exchange rate for FKBP12 was 5300 ± 300 s^{-1} and that for ubiquitylated FKBP12 K53C was 3200 ± 200 s^{-1}. Global fitting was performed using the program GLOVE[7]. Errors were calculated by the Monte Carlo method[7]. **b**, Mapping of the residues with ^{15}N relaxation dispersion for both (non-)ubiquitylated FKBP12 onto the structure of FKBP12 (PDB database accession code: 2PPN).

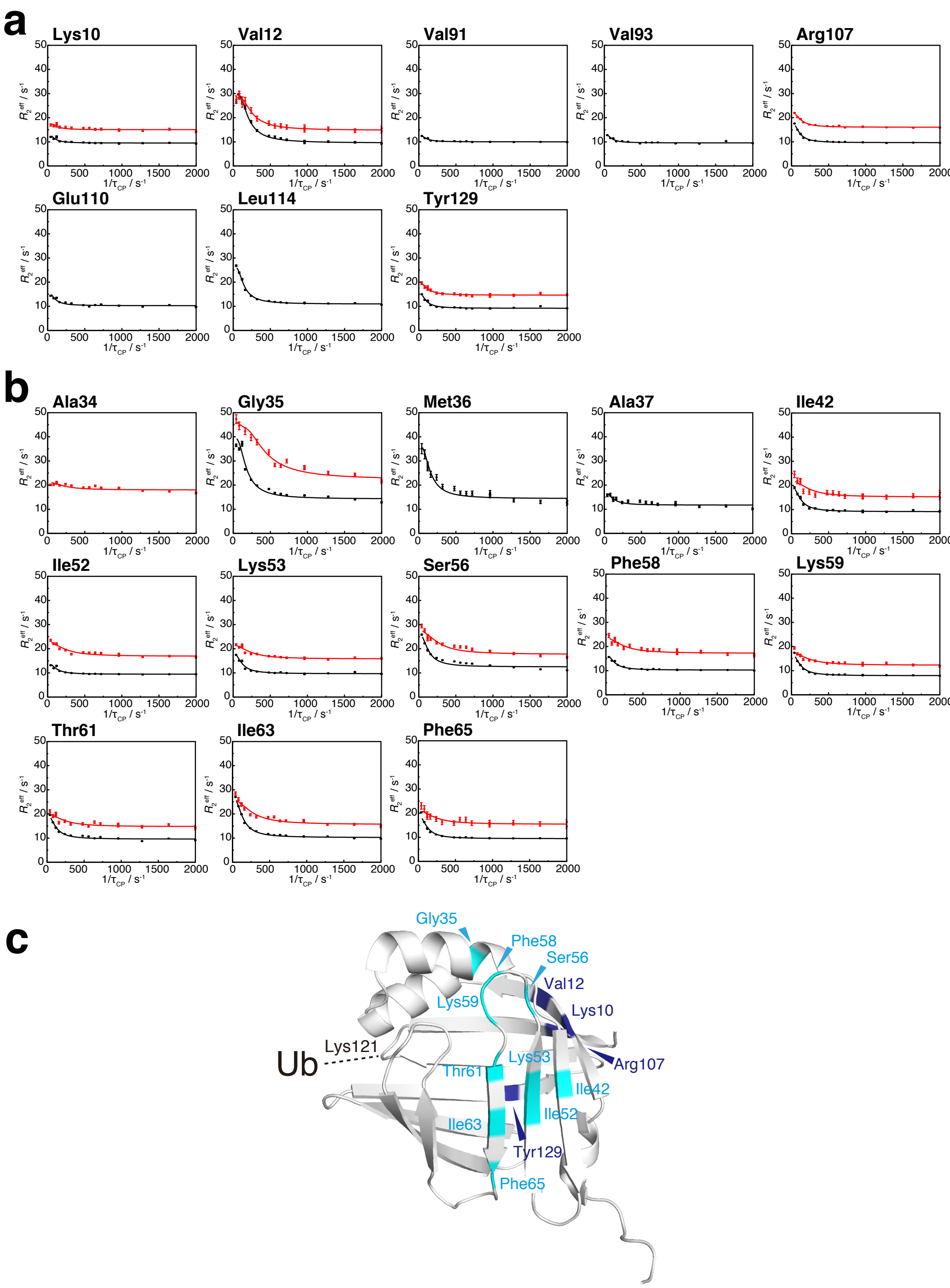

Figure S10. Relaxation dispersion analysis for ubiquitylated FABP4. ^{15}N relaxation dispersion profiles for FABP4 K121C (black) and chemically ubiquitylated FABP4 via C121 (red). ^{15}N R_2 relaxation rates were measured by the Carr-Purcell-Meiboom-Gill (CPMG) pulse sequence[5]. The effective R_2 relaxation rate at a particular delay τ_{CP} was calculated as Figure S10. Respective data were globally fitted to the Carver and Richards equation[8]. FABP4 is suggested

to possess multiple structural motions[9]: the two main motions of them are the motion related with the ligand binding in the cavity (**a**) and the motion for ligand entry on the portal region (**b**). Therefore, the data were divided into the two clusters (**a** and **b**) and analyzed differently. The obtained exchange rates for FABP4 K121C were 272 ± 4 s^{-1} (**a**) and 344 ± 4 s^{-1} (**b**). The populations of the minor states for FABP4 K121C were 8.7 ± 0.2 % (**a**) and 10.6 ± 0.2 % (**b**). On the other hand, the exchange rates for ubiquitylated FABP4 were 317 ± 2 s^{-1} (**a**) and 715 ± 36 s^{-1} (**b**). The populations of the minor states for ubiquitylated FABP4 were 5.1 ± 0.4 % (**a**) and 4.6 ± 0.3 % (**b**). Global fitting was performed using the program GLOVE[7]. Errors were calculated by the Monte Carlo method[7]. **c**, Mapping of the residues with ^{15}N relaxation dispersion for both (non-)ubiquitylated FABP4 onto the structure of FABP4 (PDB database accession code: 3RZY). The ubiquitylation site is indicated.

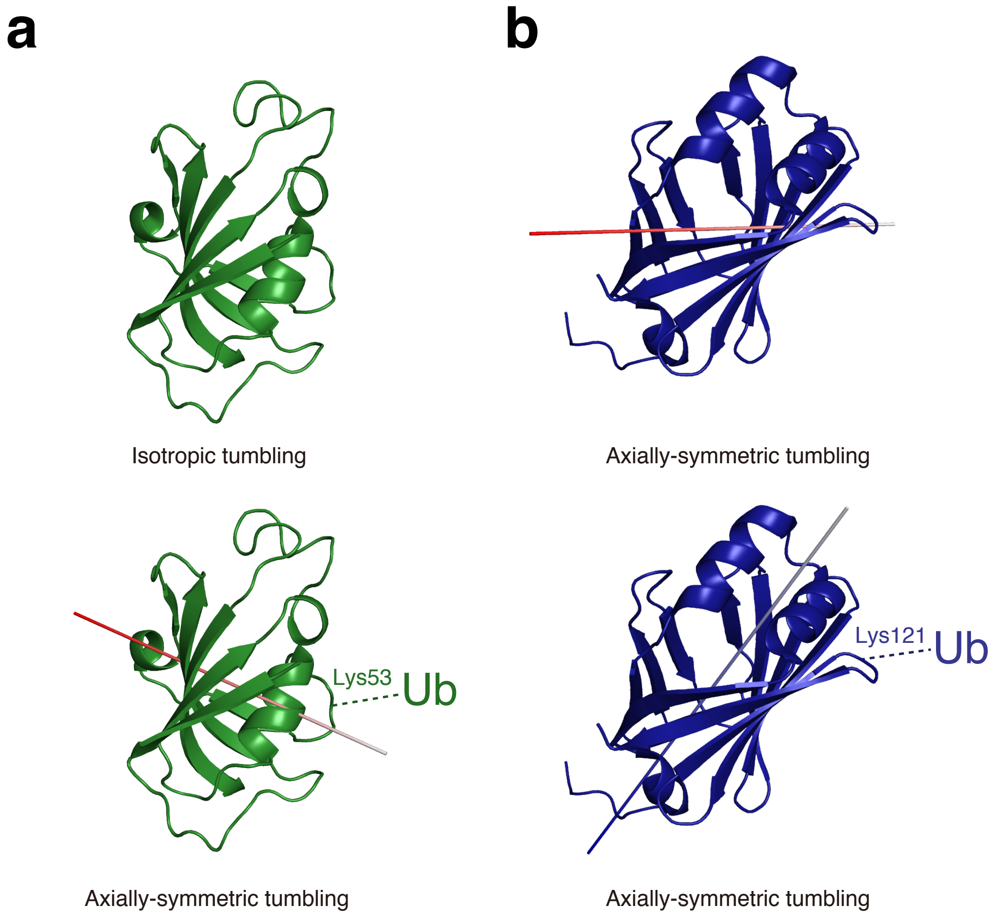

Figure S11. Ubiquitylation-induced changes in the rotational diffusion tensor. The rotational diffusion tensor of non-ubiquitylated (upper) and ubiquitylated (lower) of FKBP12 (**a**) and FABP4 (**b**) calculated from the ^{15}N T_1, T_2 relaxation times, and ^{1}H-^{15}N hetero-nuclear NOE values using the program *ROTDIF*[10]. The data of ubiquitylated FKBP12 and (non-)ubiquitylated FABP4 were fitted to the axially-symmetric model. The data of FKBP12 were fitted to the isotropic model. The red cylinders indicate individual perpendicular components of the rotational diffusion tensor ($D_{\perp}$) and the blue one indicates the parallel component of the rotational diffusion tensor ($D_{\|}$). Each tensor parameter is shown in Table S1. PDB database accession codes: 2PPN and 3RZY.

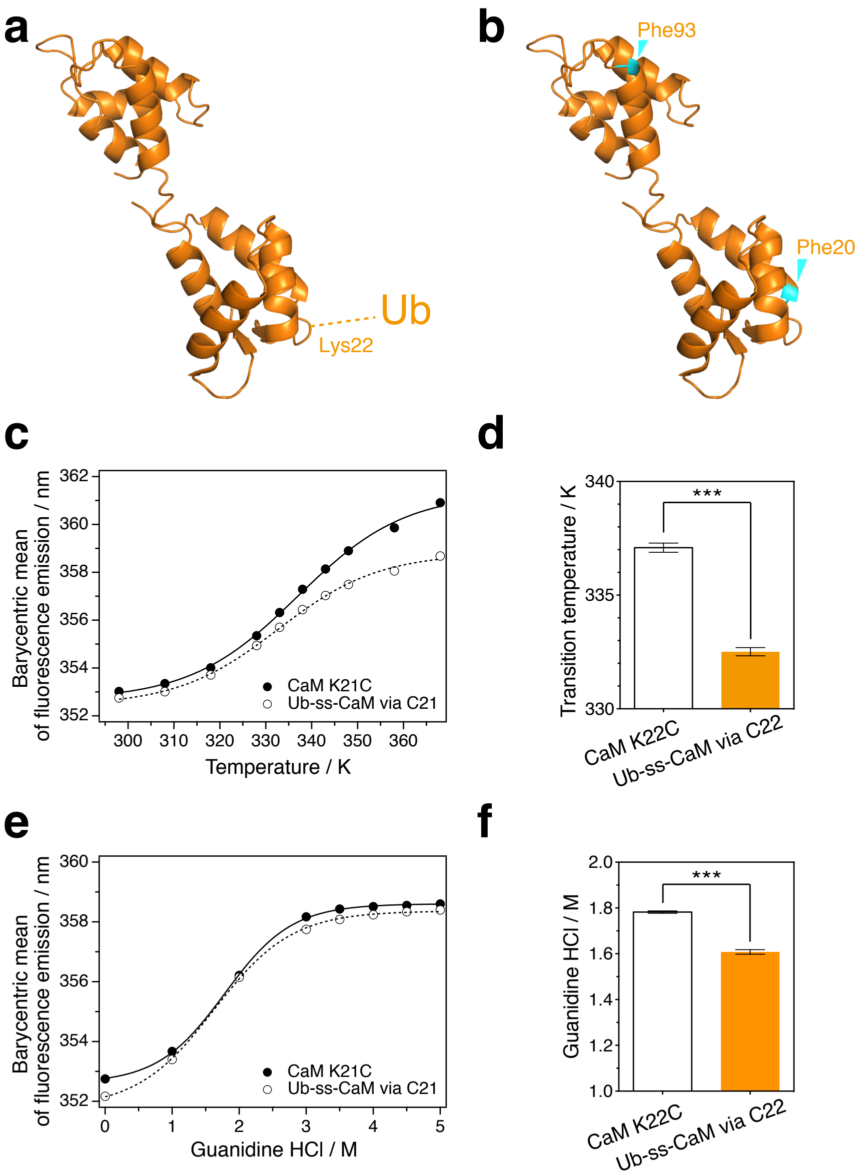

Figure S12. Fold destabilization of a multi-domain protein by ubiquitylation. a, Schematic diagram of calmodulin (CaM) ubiquitylated at Lys22. According to the PhosphoSite Plus database, CaM is ubiquitylated at Lys14, Lys22, Lys31, Lys76, Lys78, Lys95, and Lys149 *in vivo*. The effect of ubiquitylation at Lys22, located in a loop between two α-helices, on fold stability was investigated. PDB database accession codes: 1CFD[1]. **b**, Mutations of CaM introduced for tryptophan fluorescence analysis. Because CaM has no tryptophan residue, the CaM K22C mutant had two additional mutations, F20W and F93W, which have been previously established for fluorescence experiments[2,3]. Because the CaM mutant was purified using EGTA as reported previously[4], it adopts the Ca^{2+}-free conformation comprising two domains[1]. **c** and **e**,

Series of fluorescence emission wavelengths (barycentric mean) of CaM (filled circles) and chemically ubiquitylated CaM (open circles). The data were fitted to the sigmoidal equation (solid and dashed line, respectively). All samples were diluted to a final substrate protein concentration of 20 μM in PBS buffer. The proteins were thermally denatured (**c**) and guanidine hydrochloride (HCl) was used for the chemical protein denaturation (**e**). Data are representative of three independent experiments. **d** and **f**, Comparative analysis of the thermal and chemical denaturation transition for CaM and CaM ubiquitylated at Lys22. The transitions for CaM were 337.1 ± 0.2 K (**d**) and 1.78 ± 0.00 M (**f**); those for ubiquitylated CaM were 332.5 ± 0.2 K (**d**) and 1.61 ± 0.01 M (**f**). ss indicates the disulfide bridge. All values represent the average of three independent experiments. Error bars indicate the standard error of the mean. ***$P < 0.001$ (Student's *t* test).

Supplementary Table

Table S1. Rotational diffusion tensor parameters for ubiquitylated proteins.

Protein	$D_{\perp}$ / 10^7 s^{-1}	$D_{\|}$ / 10^7 s^{-1}	α / º	β / º	τ_c / ns	ζ
FKBP12	2.39 ± 0.00	2.39 ± 0.00	-	-	6.96 ± 0.01	-
Ubiquitylated FKBP12	1.40 ± 0.01	0.84 ± 0.01	47 ± 2	147 ± 2	13.75 ± 0.04	0.60 ± 0.01
FABP4	2.30 ± 0.01	1.54 ± 0.01	34 ± 1	150 ± 1	8.15 ± 0.01	0.67 ± 0.01
Ubiquitylated FABP4	1.21 ± 0.01	1.56 ± 0.01	89 ± 4	146 ± 1	12.53 ± 0.02	1.29 ± 0.02

$D_{\perp}$, $D_{\|}$ are the perpendicular, parallel components of the rotational diffusion tensor; α, β are Euler angles; τ_c is the overall correlation time; and ζ is the anisotropy.

Errors were estimated by the Monte Carlo method.

References

1 Kuboniwa, H. *et al.* Solution structure of calcium-free calmodulin. *Nat Struct Biol* **2**, 768-776 (1995).

2 Black, D. J., Tikunova, S. B., Johnson, J. D. & Davis, J. P. Acid pairs increase the N-terminal Ca2+ affinity of CaM by increasing the rate of Ca2+ association. *Biochemistry* **39**, 13831-13837 (2000).

3 Black, D. J. *et al.* Calmodulin interactions with IQ peptides from voltage-dependent calcium channels. *Am J Physiol Cell Physiol* **288**, C669-676, doi:10.1152/ajpcell.00191.2004 (2005).

4 Morimoto, D. *et al.* The unexpected role of polyubiquitin chains in the formation of fibrillar aggregates. *Nat Commun* **6**, 6116, doi:10.1038/ncomms7116 (2015).

5 Loria, J., Rance, M. & Palmer, A. A relaxation-compensated Carr-Purcell-Meiboom-Gill sequence for characterizing chemical exchange by NMR spectroscopy. *Journal of the American Chemical Society* **121**, 2331-2332, doi:10.1021/ja983961a (1999).

6 Luz, Z. & Meiboom, S. Nuclear Magnetic Resonance Study of the Protolysis of Trimethylammonium Ion in Aqueous Solution—Order of the Reaction with Respect to Solvent. *Journal of Chemical Physics* **39**, 366-&, doi:10.1063/1.1734254 (1963).

7 Sugase, K., Konuma, T., Lansing, J. C. & Wright, P. E. Fast and accurate fitting of relaxation dispersion data using the flexible software package GLOVE. *J Biomol NMR* **56**, 275-283, doi:10.1007/s10858-013-9747-5 (2013).

8 Carver, J. & Richards, R. A general two-site solution for the chemical exchange produced dependence of T_2 upon the carr-Purcell pulse separation. *Journal of Magnetic Resonance* **6**, 89-&, doi:10.1016/0022-2364(72)90090-X (1972).

9 Hotamisligil, G. S. & Bernlohr, D. A. Metabolic functions of FABPs--mechanisms and therapeutic implications. *Nat Rev Endocrinol* **11**, 592-605, doi:10.1038/nrendo.2015.122 (2015).

10 Berlin, K., Longhini, A., Dayie, T. K. & Fushman, D. Deriving quantitative dynamics information for proteins and RNAs using ROTDIF with a graphical user interface. *J Biomol NMR* **57**, 333-352, doi:10.1007/s10858-013-9791-1 (2013).
